# Silence of the Forest

**Published:** 1874-04

**Authors:** 


### SILENCE OF THE FOREST.

WE often read in books of travel of the silence and gloom of the Brazilian forests. They are realities, and the impression deepens on a longer acquaintance. The few sounds of birds are of that pensive or mysterious character which intensifies the feeling of solitude rather than imparts a sense of life and cheerfulness. Sometimes, in the midst of the stillness, a sudden yell or scream will startle one: this comes from some defenceless fruit eating animal, which is pounced upon by a tiger cat or stealthy boa constrictor. Morning and evening the howling monkeys make a most fearful and harrowing noise, under which it is difficult to keep up one's buoyancy of spirit. The feeling of inhospitable wildness which the forest is calculated to inspire is increased tenfold under the fearful uproar. Often, even in the still hours of midday, a sudden crash will be heard resounding afar through the wilderness, as some great bough or entire tree falls to the ground. There are, besides, many sounds which it is impossible to account for. I found the natives generally as much at a loss in this respect as myself. Sometimes a sound is heard like the clang of an iron bar against a hard, hollow tree, or a piercing cry rends the air; these are not repeated, and the succeeding silence tends to heighten the unpleasant impression which they make on the mind. With the natives it is always the Curupira, the wild man or spirit of the forest, which produces all noises they are unable to explain. Myths are the rude theories which mankind, in the infancy of knowledge, invent to explain natural phenomena. The Curupira is a mysterious being, whose attributes are uncertain, for they vary according to locality. Sometimes he is described as an ourang outang, being covered with long, shaggy hair, living in trees; at others he is said to have cloven feet and bright red face. He has a wife and children, and sometimes comes down to the roças to steal the mandioca. At one time I had a Mameluco youth in my service whose head was full of the legends and superstitions of the country. He always went with me into the forest; in fact, I could not get him to go alone, and whenever we heard any of the strange noises mentioned above he used to tremble with fear. He would crouch down behind me and beg of me to turn back. He became easy only after he had made a charm to protect us from the Curupira. For this purpose he took a young palm leaf, plaited it, and formed it into a ring, which he hung to a branch on our track.

